# 

**Published:** 1905-02

**Authors:** 


### CONTENTS.

ORIGINAL COMMUNICATIONS—

FACTS AND FALLACIES IN GASTRO-INTESTINAL WORK. By L. Amster, M.D., Atlanta, Ga. .......... 721

PROSTATIC MASSAGE. By W. L. Champion, M.D., Atlanta, Ga. .......... 729

IS RADIO-THERAPY A FAD? .......... 731

SOCIETY REPORTS—

CLINICAL SOCIETY OF THE NEW YORK POLYCLINIC MEDICAL SCHOOL AND HOSPITAL .......... 733

EDITORIAL NOTES AND COMMENTS—

SAMPLE AND CIRCULAR ADVERTISING—TRAVELING REPRESENTATIVES .......... 745

THE ORIGIN OF SYPHILIS .......... 747

THE GEORGIA STATE COMMISSION ON TUBERCULOSIS .......... 748

NEWS AND NOTES .......... 749

CONTENTS CONTINUED .......... X

### *CONTENTS—CONTINUED.*

ELECTIONS AND ABSTRACTS—

The Choice and Use of Medical Literature ........ 751
Scarlet Fever ........ 760
Advice to Young Doctors—Should the Young Doctor Marry? ........ 764
A Few Thoughts on Medical Life Insurance ........ 767
The Strange Case of the Rev. Mr. Hanna ........ 776
The Etiology, Prevention and Blood Serum Diagnosis of Cancer of the Stomach and Intestines ........ 781
Lithemic Naso-Pharyngitis Due to Systematic Disturbance ........ 783
The New Pharmacopeia ........ 783
The Different Systems of Medical Practice ........ 785
The Dishonest Druggist ........ 786
Kress & Owen *vs.* Crittenden ........ 787
Dairyman Held Responsible for Typhoid Germs in Milk ........ 788
Wood Sent to Jail ........ 788
Identification by Teeth Casts ........ 789
The Prevention of Smallpox ........ 789
Conservatism ........ 790
Biography of a Fool ........ 790

OOK REVIEWS ........ 791

IEDICAL ITEMS—CONTINUED ........ xvi, xviii, xx, xxii, xxiv, xxvi

